# Female Employees’ Perception of Breastfeeding Support in the Workplace, Public Universities in Spain: A Multicentric Comparative Study

**DOI:** 10.3390/ijerph17176402

**Published:** 2020-09-02

**Authors:** Águeda Cervera-Gasch, Desirée Mena-Tudela, Fatima Leon-Larios, Neus Felip-Galvan, Soukaina Rochdi-Lahniche, Laura Andreu-Pejó, Víctor Manuel González-Chordá

**Affiliations:** 1 Department of Nursing, Faculty of Health Sciences, Universitat Jaume I, Avda. Sos I Baynat s/n, 12071 Castellón, Spain; cerveraa@uji.es (Á.C.-G.); al365050@uji.es (N.F.-G.); al362118@uji.es (S.R.-L.); pejo@uji.es (L.A.-P.); vchorda@uji.es (V.M.G.-C.); 2 Nursing Department, Faculty of Nursing, Physiotherapy and Podiatry, University of Seville, 41009 Seville, Spain; fatimaleon@us.es

**Keywords:** breastfeeding, workplace, female employment, lactation, breastfeeding support, breastfeeding barrier

## Abstract

Background: Despite scientific recommendations for exclusive breastfeeding until 6 months of age and complementary breastfeeding to 2 years of age, breastfeeding abandonment rates increase with time, and one of the main reasons is that women go back to work. Aim: To analyze the perception of support of breastfeeding workers to continue breastfeeding at two Spanish universities, and associated factors. Methods: A multicenter retrospective cross-sectional comparative study conducted in a population of 777 female workers at the Universidad de Sevilla (US) and the Universitat Jaume I (UJI) in Spain using an online questionnaire. Results: The response rate was 38.74% (*n* = 301). Of all the participants, 57.8% continued breastfeeding after returning to work. The factors associated with continuing breastfeeding for longer were the university having a breastfeeding support policy and special accommodation (*p* < 0.001); participating in breastfeeding support groups (*p* < 0.001); intending to continue breastfeeding after returning to work (*p* < 0.001); knowing the occupational legislation in force (*p* = 0.009); having a female supervisor (*p* = 0.04). Conclusion: Breastfeeding support initiatives and having special accommodation to pump and preserve breast milk after returning to work are associated with a longer duration of female workers’ breastfeeding.

## 1. Introduction

The World Health Organization (WHO) recommends exclusive breastfeeding for the first 6 months of life, introducing appropriate safe foods for the baby’s age, and maintaining breastfeeding until 2 years, if the breastfed infant still wants breast milk [1]. The newborn’s first year of life is the period of fastest growth and physical development, which is why the nutritional demands during this period are so high. During this period, breast milk as a first natural food provides all the nutrients and energy required to cover a baby’s requirements [2]. Breast milk also positively affects breastfed infants’ and mothers’ health [3,4,5].

Notwithstanding, international breastfeeding rates fall after birth. The United Nations Children’s Fund (UNICEF) indicates that only 43% of newborns continue to receive exclusive breastfeeding at 6 months of life [3,6]. In Spain, a recent study indicates that the breastfeeding rate at 6 months of life is 16.8% [7]. These figures are far removed from the world objective set out by the WHO in the Comprehensive Implementation Plan on Maternal, Infant and Young Child Nutrition for 2025: increasing the exclusive breastfeeding rate for the first 6 months of life to at least 50% [8].

In Spain, maternity leave is established at 16 weeks [9]. After returning to work, mothers have one paid hour a day to breastfeed their infant until he or she is 9 months old [9]. Unpaid childcare leave can be extended up to the infant’s third birthday. All this is reflected in the Spanish law of 2009.

Women returning to work seriously affects breastfeeding rates 4 months after the baby’s birth [7,10], which is the second reason for abandoning breastfeeding [11]. The main reason why the mothers who return to work interrupt breastfeeding is the work–family conciliation challenge [12]. The challenge of work–family conciliation is for companies to create the appropriate conditions for men and women to develop their respective roles, both in the family and in the workplace. Work–life balance has a direct impact on the attitudes and behaviors of workers, improving results [13,14].

The need to adopt policies that defend work–family conciliation and support breastfeeding is fundamental, and legislation and financial investment are necessary [15] for this conciliation to become a reality. Changing and supporting these policies by employers appears to be effective in maintaining breastfeeding [16]. Such policies do not appear to have been widely adopted in Spain [14,16]. Recently, the Spanish Ministry of Health and the Baby-Friendly Hospital Initiative (BFHI) reached an agreement to promote, protect and support breastfeeding by promoting environments that favor it; e.g., creating mother-friendly places for women who decide to continue breastfeeding after going back to work [17]. However, no literature has been found that shows results from these changes.

A recent systematic review shows evidence that breastfeeding support programs and support policies at work can positively influence breastfeeding beyond maternity leave [18]. However, studies also agree on the need to evaluate if such interventions prolong breastfeeding duration after returning to work [16,19].

The main objectives of this study were to: (a) analyze mothers’ perception of breastfeeding support from both the organization and coworkers after returning to work at two Spanish public universities; (b) establish which variables predict breastfeeding duration at university.

## 2. Materials and Methods

### 2.1. Design

A multicenter retrospective cross-sectional comparative study took place between January 2017 and April 2020. For the study design, the STROBE statement for cross-sectional studies was applied [20].

A questionnaire was sent with a letter of presentation to inform about the study objectives, and to indicate that the study was voluntary, confidential and anonymous. The Ethics Committee of the Universidad de Sevilla (17102/2016) and the Deontological Committee of the Universitat de Jaume I (CD/49/2019) approved this study. The Declaration of Helsinki principles were respected. Spanish Organic Law 3/2018, of 5 December, on Personal Data Protection and the Spanish Guarantee of Digital Rights, was met.

### 2.2. Setting

The study took place at two Spanish public universities from Andalusia (Universidad de Sevilla–US–south Spain) and the Valencian Community (Universitat Jaume–UJI-east Spain).

The UJI has four specific areas designated for breastfeeding in different faculties. Since 2006, meetings with breastfeeding mothers have been held at the UJI. These sessions are organized and led by the local breastfeeding support group, which deals with matters like the benefits of breastfeeding, and pumping/preserving/storing breast milk and experiences. They last approximately 3 h. At the time this article was written, the US had neither a breastfeeding room, nor any program to support or promote breastfeeding.

### 2.3. Sample

In 2016, the US had 6652 employees (4120 teachers/researchers (PDI) and 2532 administration/services staff (PAS)). Of these, 37.67% (*n* = 1552) were female PDI and 48% (*n* = 1215) were female PAS [21]. The UJI had 2195 employees (1542 PDI and 653 PAS), of whom 47.28% (*n* = 729) were female PDI and 60.64% (*n* = 396) were female PAS [22].

The inclusion criteria of our sample were: female, of age, employed at the US or the UJI (PAS or PDI) and having given birth in the past 10 years. The exclusion criteria were women who did not work for either university when they gave birth and/or breastfed, and not completing questionnaires properly. A sample of 107 participants was estimated to be necessary by assuming both an infinite population and maximum indetermination hypothesis, a 95% confidence level, 10% accuracy and a 10% replacement rate.

### 2.4. Measurement

The questionnaire included socio-demographic variables, workplace (PAS-PDI), gyneco-obstetric variables (including birth experience on a Likert scale from 1: very bad to 5: marvelous), intention to continue breastfeeding (yes/no), continuing breastfeeding when back at work (yes/no) and mothers perceiving breastfeeding support at their workplace. Mothers’ perception was measured with the validated Workplace Breastfeeding Support Scale (WBSS) [23]. The WBSS version employed was that validated for the Spanish context [12], with 18 items measured on a Likert scale from 1 to 7 (1: strongly disagree, 7: strongly agree) grouped into four categories: Technical Support, Environmental Support, Break Time; Workplace Policy. This scale was answered by those women who continued breastfeeding after returning to work (Supplementary Materials). For this study, the Cronbach’s alpha of this scale’s internal consistency was 0.840.

### 2.5. Data Collection

Data collection was performed with an online questionnaire created with Google Drive™, which was sent to all the female workers who had become mothers in the past 10 years. This questionnaire was distributed by the Central Services of the two universities to all the working mothers of their university community via email. Data collection also involved consecutive case sampling to complete the required sample size. The questionnaires that met the inclusion criteria were selected.

### 2.6. Data Analysis

Data were processed by SPSS v.25 (IBM, Armonk, NY, USA). A descriptive analysis was done of all the variables. For the quantitative variables, the mean, standard deviation, maximum and minimum were calculated, while the distribution of frequencies and percentages were calculated for the qualitative variables. For the bivariate analysis, chi-squared, Mann–Whitney U and Kruskal–Wallis tests were run according to the applicability conditions (Kolmogorov–Smirnov < 0.05).

A multivariate analysis was performed by ordinal logistic regression. The maximum likelihood method was employed, and the complementary log–log link method was followed because the higher category of the dependent variable was expected to be the most frequent (longer breastfeeding duration).

Owing to the low frequency of some multivariate analysis categories, the following variables were categorized: birth weight (<3000 g; >3000 g); birth type (vaginal/cesarean); birth experience (less than or equal to 3 points; more than 3 points); breastfeeding duration (less than 6 months/1–2 years/more than 2 years); time maternity leave lasted (less than 16 weeks/more than 16 weeks). Women currently breastfeeding were excluded from this analysis.

A first regression was done by including all the socio-demographic and gyneco-obstetric variables considered in this study to calculate the Wald z statistic and to determine the dependence of variables. Next, a new regression was done that included the statistically significant independent variables in at least one dependent variable category. Goodness of fit was explored by the likelihood ratio G statistic and Pearson’s chi-squared and chi-squared test of deviations. The employed determination coefficient to study the explained variance was Nagelkerke pseudo-R2 (acceptable value R2 ≈ 0.5). The parallel lines test was carried out to verify the suitability of the coefficients of the independent variables and to determine the model’s feasibility. Finally, in order to evaluate the different models’ predictive capacity, the predicted categories were stored in the database and the index of similarity (observed agreement) was obtained with contingency tables. The level of significance was taken as *p* < 0.05.

## 3. Results

From both universities, 777 (US = 556, UJI = 221) female workers were eligible to participate in this study. The response rate was 38.74% (*n* = 301; US: 35.6%, *n* = 198; UJI: 46.6%, *n* = 103) and there was no access to, or records for, their reasons for not participating. The final study sample included 301 women. Their mean age was 41.94 years (SD = 4.55, Min = 31, Max = 53; *p* = 0.333). Of them all, 91.7% (*n* = 276) had completed university studies, 46.2% (*n* = 139) were PAS and the rest were PDI (53.8%; *n* = 162). Of the whole sample, 17.3% (*n* = 52) had problems during their last pregnancy, with gestational diabetes (28.9%, *n* = 15), possible premature birth (13.5%, *n* = 7), possibility of spontaneous miscarriage (13.5%, *n* = 7) and preeclampsia (11.5%, *n* = 6) being the most frequent problems. Babies’ mean birth weight was 3.27 kg (SD = 0.56, Min= 0.71, Max = 5.3; *p* = 0.992). Birth experience obtained a mean score of 3.87 points (Min = 1, Max = 5; *p* = 0.016). Table 1 provides a comparison of the samples between both universities, which were homogeneous.

On the whole, 87.4% (*n* = 263) breastfed all their children. Of those female workers who did not breastfeed all their children (*n* = 38, 12.6%), the main reasons were hypogalactia (47.4%, *n* = 18), own decision (13.2%, *n* = 5) and health problems (13.2%, *n* = 5). Information about breastfeeding during pregnancy was received by 90% (*n* = 271) and 96.3% (*n* = 290) intended to breastfeed their infant while pregnant. Intention to continue breastfeeding when back at work was reported by 70.4% (*n* = 212), and 57.8% (*n* = 174) continued breastfeeding once back at work. Table 2 offers the results of the breastfeeding-related variables and the family–work conciliation measures compared by sample.

### 3.1. Results of the Breastfeeding Duration Variables

The socio-demographic variables showing statistical significance for breastfeeding duration were: the university (US: M = 1.98, SD = 1.080; UJI: M = 2.86, SD = 1.095, *p* < 0.001); having participated in a breastfeeding support group (Yes: M = 3.05, SD = 0.999, No = 2.03, SD = 1.03; *p* < 0.001); intending to continue breastfeeding once back at work (Yes: M = 2.58, SD = 1.107; No: M = 1.28, SD = 0.696; *p* < 0.001); continuing breastfeeding when back at work (Yes: M = 2.82, SD = 1.029; No: M = 1.34, SD = 0.699; *p* < 0.001); knowing the breastfeeding legislation currently in force (Yes: M = 2.17, SD = 1.200; No: M = 2.40, SD = 1.118; *p* = 0.009); supervisor’s gender (Man: M = 2.32, SD = 1.159; Woman: M = 2.26, SD = 1.174; *p* = 0.040).

### 3.2. The Workplace Breastfeeding Support Scale (WBSS) Results

The mean general score obtained for the WBSS scale was 3.61 points (SD = 1.060, 95%CI = 3.45–3.78). For all the dimensions, the general score was: mean of 3.82 points (SD = 1.602, 95%CI = 3.57–4.08) for the Break Time dimension; mean of 3.95 points (SD = 1.218, 95%CI = 3.75–4.14) for the Environmental Support dimension; the mean score for the Technical Support dimension was 2.59 points (SD = 1.453, 95%CI = 2.36–2.82); the mean score for the Workplace Policy dimension was 3.61 points (SD = 1.060, 95%CI = 3.45–3.78).

The highest scoring items were: “My coworkers do not make fun of me when I sometimes leak milk through my clothes” (M = 5.01, SD = 1.964, 95%CI = 4.70–5.32); “My coworkers agree that breastfeeding is better for baby’s health than formula feeding” (M = 4.66, SD = 1.707, 95%CI = 4.39–4.93); “I would feel comfortable asking for accommodation to help me breastfeed or pump breast milk at work” (M = 4.54, SD = 2.007, 95%CI = 4.22–4.85) and “My coworkers listen to me talk about my breastfeeding experience” (M = 4.44, SD = 1.820, 95%CI = 4.15–4.73). The items with the lowest scores for the general outcomes were: “My workplace has a breast pump for nursing mothers to use” (M = 1.39, SD = 1.072, 95%CI = 1.22–1.56); “My job could be at risk (e.g., lose my job or get fewer scheduled hours) if I breastfed or pumped breast milk at work” (M = 2.45, SD = 2.014, 95%CI = 2.13–2.77); “Breastfeeding is common in my workplace” (M = 2.48, SD = 1.759, 95%CI = 2.20–2.76) and “I can easily find a quiet place other than the bathroom at work to pump breast milk” (M = 2.97, SD = 2.106, 95%CI = 2.63–3.30).

The WBSS scale results are shown in Table 3 according to job post (PAS/PDI), in Table 4 according to the university where the women worked and Table 5 according to the occupational situation at each university.

### 3.3. Multivariate Analysis Results

Generally speaking, with the first regression results, and by including all the socio-demographic and gyneco-obstetric variables, the parallel lines test did not confirm that the estimations were the same for all the variables of the dependent category (*p* < 0.001). Those variables whose categories showed statistical significance to be included in the second regression were: university, having a partner, attended antenatal classes, received breastfeeding information, time maternity leave lasted, participated in breastfeeding support groups and continued breastfeeding once back at work (*p* < 0.05) (Table 6).

In the second regression, the overall fit test with G statistic confirmed that the model improved when variables were included as opposed to the model with only the constant (chi-squared = 147.044; *p* < 0.01). The model’s goodness of fit was confirmed with Pearson’s chi-squared (chi = 134.667; *p* = 0.055) and the chi-squared test of deviations (chi = 113.651; *p* = 0.387). The Nagelkerke pseudo-R2 obtained a value of 0.480 and the parallel lines test confirmed that the ß coefficients were the same for all the independent variables categories (chi-squared = 3.930; *p* = 0.996). The model had a predictive capacity of 34.0% (*p* < 0.001), and correctly predicted 37.5% (*n* = 93) of the cases with breastfeeding for less than 6 months, 33.9% (*n* = 84) of the cases continuing breastfeeding for 1–2 years and 28.6% (*n* = 71) of the cases of breastfeeding for more than 2 years.

## 4. Discussion

This study aimed to compare how working mothers perceived breastfeeding support at Spanish public universities once they had returned to work. Our intention was to compare the perceptions of those mothers working for an institution with a pro-breastfeeding policy, rooms for pumping breast milk and periodic breastfeeding support group sessions as opposed to those mothers working for an institution with no such support in their workplace. This study also allowed us to detect which factors contributed to breastfeeding duration in these contexts.

The main findings obtained in the present research work showed that differences existed between the two universities that adopted distinct breastfeeding support policies. The experiences of the female workers working at the university offering accommodation to pump breast milk led them to give better scores for continuing to breastfeed, which agrees with Kozhimannil, Jou, Gjerdingen and McGovern [24]. The above-cited authors discovered that those women with access to rooms where they could pump breast milk and take breaks were more likely to continue with exclusive breastfeeding at 6 months than those with no access to such accommodation [24]. In line with this result, it is worth pointing out that, as reported by the studies of Lee, Chang and Chang [25] and Nabulsi et al. [26], those women who continued breastfeeding longer had attended breastfeeding support groups [27].

As with other previous studies [10,12], the breastfeeding rate after going back to work dropped considerably, which is a critical breastfeeding abandonment factor in Spain [7,28].

There are various scales for assessing return to work and breastfeeding. The scale developed by Bar-Yam [29] is primarily aimed at helping mothers prepare for a return to work. The WBSS aims to assess the degree of breastfeeding support in the workplace after a mother’s return to work [30]. Bai et al. proposed expanding the scale by adding elements reflecting various aspects of technical support such as having separate refrigerators for breast milk, hand washing facilities or access to breast pump outlets [23]. In the most recent version of the scale, it was expanded, this being the one used in the Spanish context [12,23].

The scale showed statistically significant differences between the universities in terms of the presence of on-site day care and supportive policies in relation to breastfeeding. The UJI scored higher on both items because, since 2006, it built a breastfeeding support policy linked to a local breastfeeding support group. The UJI also has an on-site day care on its premises. The US lacked such support. Those women who worked at the university with breastfeeding support policies perceived better breastfeeding support and continued this practice longer. Other studies confirmed that women open to breastfeeding support programs at work reported increases in the initiation of breastfeeding, exclusive breastfeeding and continuing this practice [27,31]. It is worth considering that comprehensive breastfeeding programs have been proven to be effective in continuing breastfeeding at the workplace, a fact that was also herein confirmed because the university that had set up support programs had higher continued breastfeeding rates [16].

Setting up a breastfeeding-friendly environment favors workers’ fidelity and satisfaction, which have positive effects on continuing breastfeeding and also influence other personal factors related to mothers and their motivation [32]. In this study, the following personal factors stood out: having a partner [33]; having attended antenatal classes [34]; having received breastfeeding information [26]; time that maternity leave lasted [35]; attended breastfeeding support groups [25,26]; continuing breastfeeding when back at work.

Although women perceived more support at the university that had set up breastfeeding support actions/breastfeeding rooms, it was noteworthy that the items belonging to the dimensions “Environmental Support” and “Break Time” showed no statistically significant differences between both universities. Moreover, the dimension “Break Time” and its items provided valuable information about women’s occupational situation at university. All this information suggests that, although providing accommodation for pumping milk is important, regulating access and times for this practice is also essential [32,36]. The professional profile of those mothers whose experience was more gratifying was found in the group of females belonging to the PDI group. These women found time more easily to pump breast milk and to, thus, ensure milk production over time, while the PAS staff members obtained worse results, which coincides with other studies [37]. As the review by Hirani and Karmaliani [38] suggests, it is necessary to conduct breastfeeding promotion programs, but these programs also need to consider occupational situations, employers and workplaces. Moreover, making working hours and breaks flexible seems to help prolong breastfeeding when mothers go back to work [39,40].

As similar studies have verified, support from coworkers and supervisors in the workplace is a factor that favors breastfeeding being prolonged [36,41] because it helps more women to opt for breastfeeding when they return to work [10].

As a study limitation, we compared two samples from different institutions, although both were homogenous and comparable. On the other hand, the homogeneity of the sample in variables such as educational level and number of children may have influenced the results of the multivariate analysis and, for this reason, they were not significant in this study. In addition, future studies should consider variables that influence breastfeeding outcomes, such as previous breastfeeding experience and the seasonality of the start of breastfeeding in relation to the academic year. The relationship between the duration of breastfeeding and reasons for quitting was also not studied and should be considered in future studies. Analyzing recent births and comparing them with older ones in such a long cohort study may be another analysis to consider in the future. Moreover, we acknowledge that a memory bias might emerge because our study was retrospective. Participation was voluntary and the response rate was acceptable, but not very high. This made us think that perhaps the most extreme opinions would be represented as those female workers who had an excellent or a bad experience could have felt more motivated to complete the questionnaire.

Finally, this study cannot be generalized to other countries with different occupational systems for female workers at universities and with very different maternity leaves. Nonetheless, we want to point out that we did not re-cover studies comparing this issue in two Spanish public universities. Despite being public bodies with a certain level of self-regulation, it would be expected that this type of institution would be at the forefront in the development of this type of policy. We believe that it is representative of what occurs at a state level and its results are interesting. Furthermore, we did not find other articles related to this topic that applied ordinal logistic regression as an analysis technique. Therefore, we believe that the results of this study are of interest to the scientific community and decision-makers.

## 5. Conclusions

Breastfeeding support policies in the workplace positively impact mothers continuing breastfeeding when they go back to work. The fact that female employees have accommodation to pump breast milk and the breaks needed to do so is positively valued by women and is associated with a longer breastfeeding when they go back to work. In the university setting, as PDI staff members have more possibilities of enjoying breaks, it is necessary to develop support policies for such breaks to include all groups of workers.

## Figures and Tables

**Table 1:** Socio-demographic and gyneco-obstetric data of the female sample (*n* = 301).

	Universidad de Sevilla (US; *n* = 198)		Universitat Jaume I (UJI; *n* = 103)		*p*-Value ^1^
	*n*	%	*n*	%	
Nationality					0.677
Spanish	193	97.5	102	99	
North American	2	1	0	0	
Italian	1	0.5	1	1	
French	1	0.5	0	0	
Swiss	1	0.5	0	0	
Level of Education					0.004
Primary education	2	1	0	0	
Secondary education	22	11.1	1	1	
University studies	174	87.9	102	99	
Job post					0.808
Administration/Services Personnel (PAS)	90	45.5	49	47.6	
Teacher/Researcher (PDI)	108	54.5	54	52.4	
Have a partner					0.230
Yes	184	92.9	102	99	
No	14	7.1	1	1	
Number of children					0.001
1	44	22.2	44	42.7	
2	115	58.1	50	48.5	
3	33	16.7	8	7.8	
4	6	3	1	1	
Health problems during pregnancy					0.770
No	158	79.8	91	88.3	
Yes	40	20.2	12	11.7	
Last birth					0.443
Cesarean for fetal emergency	21	10.6	17	16.5	
Scheduled cesarean	28	14.1	17	16.5	
Natural birth	107	54	44	42.7	
Induced birth	17	8.6	10	9.7	
Instrumented birth	24	12.1	15	14.6	
Attended antenatal classes					0.088
No	34	17.2	10	9.7	
Yes	164	82.8	93	90.3	

^1^ Chi-squared test with Fisher’s correction whenever necessary.

**Table 2:** Data on breastfeeding and family–work conciliation measures (*n* = 301).

	Universidad de Sevilla (US; *n* = 198)		Universitat Jaume I (UJI; *n* = 103)		*p*-Value ^1^
	*n*	%	*n*	%	
You breastfed your children					0.823
No	14	7.1	7	6.8	
Yes, all of them	174	87.9	89	86.4	
Yes, but not all of them	10	5.1	7	6.8	
Breastfeeding information					0.226
No	23	11.6	7	6.8	
Yes	175	88.4	96	93.2	
Intention to breastfeed					0.051
No	4	2.0	7	6.8	
Yes	194	98.0	96	93.2	
Breastfeeding last-born child					0.837
No	18	9.1	10	9.7	
Yes	180	90.9	93	90.3	
Attended a breastfeeding support group					<0.001
No	164	82.8	40	38.8	
Yes	16	8.1	53	51.5	
Intention to continue breastfeeding when back at work					0.580
No	48	24.4	13	12.6	
Yes	132	66.7	80	77.7	
Continued breastfeeding when back at work					0.001
No	78	39.4	19	18.4	
Yes	100	50.5	74	71.8	
Breastfeeding duration					<0.001
Less than 6 months	79	39.9	16	15.5	
6–12 months	36	18.2	14	13.6	
1–2 years	33	16.7	29	28.2	
More than 2 years	21	10.6	33	32.0	
Currently breastfeeding	8	4	-	-	
Maternity leave time					0.451
6 weeks	3	1.5	4	3.9	
7–11 weeks	6	3	4	3.9	
12–16 weeks	43	21.7	26	25.2	
More than 16 weeks	116	58.6	59	57.3	
Work a shorter working day					0.800
No	138	69.7	68	66	
Yes	42	21.2	25	24.3	
Knowledge of legislation					0.784
No	89	44.9	50	48.5	
Yes	91	46	43	41.7	

^1^ Chi-squared test with Fisher’s correction whenever necessary.

**Table 3:** Results obtained with the Workplace Breastfeeding Support Scale. Comparison of occupational situation (*n* = 155).

	PAS (*n* = 64)		PDI (*n* = 91)		*p*-Value ^1^
	M	SD	M	SD	
**Total**	3.35	1.006	3.80	1.061	0.013
**Break Time dimension**	3.31	1.537	4.18	1.556	0.001
My breaks are frequent enough for breastfeeding or pumping breast milk	3.09	1.998	4.30	2.095	0.001
My breaks are long enough for breastfeeding or pumping breast milk	3.13	2.035	4.31	2.133	0.001
I could adjust my break schedule in order to breastfeed or pump breast milk	3.25	2.024	4.75	2.042	<0.001
I feel comfortable taking several breaks during working hours to pump breast milk	2.50	1.727	3.43	2.146	0.009
I have supportive coworkers who cover for me when I need to pump my milk	3.39	2.150	3.76	2.228	0.337
I would feel comfortable asking for accommodation to help me breastfeed or pump breast milk at work	4.52	2.039	4.55	1.996	0.976
**Environmental Support dimension**	3.82	1.150	4.03	1.262	0.515
Breastfeeding is common in my workplace	2.30	1.580	2.60	1.873	0.469
My coworkers agree that breastfeeding is better for baby’s health than formula feeding	4.53	1.718	4.75	1.704	0.481
My supervisor says things that make me think he/she supports breastfeeding	3.95	1.794	4.24	1.797	0.523
My coworkers do not make fun of me when I sometimes leak milk through my clothes	4.98	1.972	5.03	1.969	0.955
I can easily find a quiet place other than the bathroom at work to pump breast milk	2.53	1.755	3.27	2.281	0.084
My coworkers listen to me talk about my breastfeeding experience	4.63	1.804	4.31	1.830	0.323
**Technical Support dimension**	2.40	1.395	2.72	1.485	0.195
My workplace has a refrigerator that I can use to store my milk	3.03	2.330	3.35	2.536	0.581
My workplace has a breast pump for nursing mothers to use	1.52	1.069	1.31	1.072	0.071
My workplace has an on-site day care	2.64	2.256	3.51	2.705	0.059
**Workplace Policy dimension**	3.42	1.156	3.66	1.184	0.013
My job could be at risk (e.g., lose my job or get fewer scheduled hours) if I breastfed or pumped breast milk at work	2.52	2.031	2.41	2.011	0.868
I would have enough maternity leave (paid and/or unpaid time off) to get breastfeeding started before going back to work	4.11	2.344	4.66	2.363	0.164
I am certain my company has written policies for employees that are breastfeeding or pumping breast milk	3.63	2.097	3.91	1.924	0.374

^1^ Mann–Whitney U Test.

**Table 4:** Results obtained with the Workplace Breastfeeding Support Scale. Comparison of universities (*n* = 155).

	US (*n* = 102)		UJI (*n* = 53)		*p*-Value ^1^
	M	SD	M	SD	
**Total**	3.47	0.929	3.88	1.239	0.031
**Break Time dimension**	3.71	1.508	4.04	1.764	0.321
My breaks are frequent enough for breastfeeding or pumping breast milk	3.64	2.043	4.11	2.284	0.228
My breaks are long enough for breastfeeding or pumping breast milk	3.67	2.122	4.11	2.242	0.245
I could adjust my break schedule in order to breastfeed or pump breast milk	3.95	2.164	4.47	2.127	0.185
I feel comfortable taking several breaks during working hours to pump breast milk	2.94	1.979	3.25	2.130	0.452
I have supportive coworkers who cover for me when I need to pump my milk	3.54	2.183	3.74	2.237	0.685
I would feel comfortable asking for accommodation to help me breastfeed or pump breast milk at work	4.51	2.105	4.58	1.823	0.780
**Environmental Support dimension**	3.80	1.090	4.22	1.403	0.047
Breastfeeding is common in my workplace	2.05	1.594	3.30	1.782	<0.001
My coworkers agree that breastfeeding is better for baby’s health than formula feeding	4.68	1.719	4.62	1.701	0.892
My supervisor says things that make me think he/she supports breastfeeding	4.03	1.777	4.30	1.835	0.282
My coworkers do not make fun of me when I sometimes leak milk through my clothes	5.15	1.937	4.75	2.009	0.225
I can easily find a quiet place other than the bathroom at work to pump breast milk	2.69	2.025	3.51	2.172	0.020
My coworkers listen to me talk about my breastfeeding experience	4.24	1.798	4.83	1.816	0.074
**Technical Support dimension**	2.25	1.219	3.24	1.645	<0.001
My workplace has a refrigerator that I can use to store my milk	3.03	2.511	3.58	2.307	0.100
My workplace has a breast pump for nursing mothers to use	1.19	.641	1.79	1.536	0.004
My workplace has an on-site day care	2.53	2.383	4.34	2.480	<0.001
**Workplace Policy dimension**	3.57	1.191	3.53	1.155	0.989
My job could be at risk (e.g., lose my job or get fewer scheduled hours) if I breastfed or pumped breast milk at work	2.63	2.082	2.11	1.847	0.107
I would have enough maternity leave (paid and/or unpaid time off) to get breastfeeding started before going back to work	4.64	2.311	4.04	2.433	0.152
I am certain my company has written policies for employees that are breastfeeding or pumping breast milk	3.45	1.922	4.45	1.986	0.002

^1^ Mann–Whitney U Test.

**Table 5:** Results obtained with the Workplace Breastfeeding Support Scale. Comparison of occupational situation at universities (*n* = 155).

	US (*n* = 102)				UJI (*n* = 53)				*p* ^1^
	PAS (*n* = 37)		PDI (*n* = 65)		PAS (*n* = 27)		PDI (*n* = 26)		
	M	SD	M	SD	M	SD	M	SD	
**Total**	3.15	0.895	3.66	0.903	3.62	1.099	4.16	1.335	0.005
**Break Time dimension**	3.08	1.319	4.07	1.499	3.64	1.769	4.47	1.688	0.004
My breaks are frequent enough for breastfeeding or pumping breast milk	2.81	1.808	4.11	2.032	3.48	2.208	4.77	2.215	0.002
My breaks are long enough for breastfeeding or pumping breast milk	2.86	1.932	4.12	2.103	3.48	2.155	4.77	2.178	0.003
I could adjust my break schedule in order to breastfeed or pump breast milk	2.57	1.482	4.74	2.101	4.19	2.304	4.77	1.925	<0.001
I feel comfortable taking several breaks during working hours to pump breast milk	2.19	1.488	3.37	2.103	2.93	1.960	3.58	2.283	0.035
I have supportive coworkers who cover for me when I need to pump my milk	3.30	2.080	3.68	2.244	3.52	2.276	3.96	2.218	0.745
I would feel comfortable asking for accommodation to help me breastfeed or pump breast milk at work	4.73	2.117	4.38	2.104	4.22	1.928	4.96	1.661	0.431
**Environmental Support dimension**	3.64	1.058	3.90	1.104	4.07	1.242	4.38	1.562	0.186
Breastfeeding is common in my workplace	1.92	1.498	2.12	1.654	2.81	1.570	3.81	1.877	<0.001
My coworkers agree that breastfeeding is better for baby’s health than formula feeding	4.62	1.754	4.71	1.711	4.41	1.693	4.85	1.713	0.850
My supervisor says things that make me think he/she supports breastfeeding	3.81	1.777	4.15	1.779	4.15	1.834	4.46	1.860	0.598
My coworkers do not make fun of me when I sometimes leak milk through my clothes	5.03	1.907	5.22	1.964	4.93	2.093	4.58	1.943	0.471
I can easily find a quiet place other than the bathroom at work to pump breast milk	2.08	1.422	3.03	2.236	3.15	1.994	3.88	2.321	0.021
My coworkers listen to me talk about my breastfeeding experience	4.38	1.861	4.15	1.770	4.96	1.698	4.69	1.955	0.291
**Technical Support dimension**	1.90	1.097	2.45	1.249	3.07	1.492	3.41	1.804	<0.001
My workplace has a refrigerator that I can use to store my milk	2.73	2.329	3.20	2.611	3.44	2.309	3.73	2.342	0.342
My workplace has a breast pump for nursing mothers to use	1.30	0.777	1.12	0.545	1.81	1.331	1.77	1.751	0.016
My workplace has an on-site day care	1.68	1.564	3.02	2.631	3.96	2.410	4.73	2.539	<0.001
**Workplace Policy dimension**	3.55	1.240	3.58	1.171	3.23	1.025	3.85	1.219	0.298
My job could be at risk (e.g., lose my job or get fewer scheduled hours) if I breastfed or pumped breast milk at work	3.24	2.253	2.28	1.908	1.52	1.087	2.73	2.255	0.012
I would have enough maternity leave (paid and/or unpaid time off) to get breastfeeding started before going back to work	4.41	2.339	4.77	2.303	3.70	2.334	4.38	2.531	0.305
I am certain my company has written policies for employees that are breastfeeding or pumping breast milk	3.00	1.886	3.71	1.910	4.48	2.101	4.42	1.901	0.007

^1^ Kruskal–Wallis test.

**Table 6:** Variables that had categories with statistical significance and were included in the later logistic regressions.

	Estimation	Wald	Sig.	95%CI	
				Inferior	Superior
Duration of breastfeeding					
Less than 6 months	−1.184	4.786	0.029	−2.245	−0.123
1–2 years	−0.348	0.422	0.516	−1.399	0.703
More than 2 years	0.692	1.647	0.199	−0.365	1.748
University					
Universidad de Sevilla	−0.426	5.016	0.025	−0.799	−0.053
Universitat Jaume I	0 ^1^				
Have a partner					
Yes	−1.043	0.405	0.01	−1.836	−0.25
No	0 ^1^				
Attended antenatal classes					
Yes	0.595	5.219	0.022	0.085	1.105
No	0 ^1^				
Breastfeeding information					
Yes	−0.788	5.432	0.02	−1.45	−0.125
No	0 ^1^				
Maternity leave time					
Less than 16 weeks	−0.321	3.688	0.055	−0.649	0.007
More than 16 weeks	0 ^1^				
Attended a breastfeeding support group					
Yes	0.532	5.91	0.015	0.103	0.96
No	0 ^1^				
Continued breastfeeding when back at work					
Yes	1.93	98.846	<0.001	1.549	2.31
No	0 ^1^				

^1^ This parameter is set at zero because it is redundant.

## Supplementary Materials

The following are available online at https://www.mdpi.com/1660-4601/17/17/6402/s1.
