# Supplementary material for: Female Employees’ Perception of Breastfeeding Support in the Workplace, Public Universities in Spain: A Multicentric Comparative Study
Source: Int J Environ Res Public Health. 2020 Sep 2;17(17):6402. doi: 10.3390/ijerph17176402 (PMC7504108; doi:10.3390/ijerph17176402)
Supplement: Supplementary file 1 [file ijerph-17-06402-s001.pdf]

Supplementary material

The following is the questionnaire used for data collection. This questionnaire was administered online and in Spanish. The questionnaire has been translated for the publication of this work.

Age: short answer

Nationality: short answer

Job post: (a)Administration/Services Personnel (PAS); (b) Teacher/Researcher (PDI)

Level of Education: (a) Primary education; (b) Secondary Education; (c) University studies

Have a partner: (a) Yes; (b) No

Number of children: short answer

You breastfed your children: (a) No; (b) Yes, all of them; (c) Yes, but not all of them

If your answer was not "Yes, all of them" indicate the reason: short answer

Health problems during pregnancy: (a) Yes; (b) No.

What problem did you have during your pregnancy? short answer

Last birth: (a) Cesarean for fetal emergency; (b) Scheduled caesarean; (c) Natural birth; (d) Induced birth; (e) Instrumented birth

Birth weight: short answer

Birth experience: 5-point scale from very negative to very positive.

Attended antenatal classes: (a) Yes; (b) No

Breastfeeding information: (a) Yes; (b) No

Intention to breastfeed: (a) Yes; (b) No

Breastfeeding last-born child: (a) Yes; (b) No

Breastfeeding duration (in months): short answer

Attended a breastfeeding support group: (a) Yes; (b) No

Maternity leave time (in weeks): short answer

Intention to continue breastfeeding when back at work: (a) Yes; (b) No

Continued breastfeeding when back at work: (a) Yes; (b) No

Knowledge of legislation: (a) Yes; (b) No

Work a shorter working day: (a) Yes; (b) No

Supervisor's gender: (a) Man; (b) Woman

If you will continue to breastfeed after returning to work, please answer the following questionnaire (Likert scale from 1 to 7 -1: strongly disagree, 7: strongly agree- grouped into four categories: Technical Support, Environmental Support, Break Time; Workplace Policy):

Break time dimension

My breaks are frequent enough for breastfeeding or pumping breast milk

My breaks are long enough for breastfeeding or pumping breast milk

I could adjust my break schedule in order to breastfeed or pump breast milk

I feel comfortable taking several breaks during working hours to pump breast milk

I have supportive coworkers who cover for me when I need to pump my milk

I would feel comfortable asking for accommodation to help me breastfeed or pump breast milk at work

Environmental Support dimension

Breastfeeding is common in my workplace

My coworkers agree that breastfeeding is better for baby's health than formula feeding

My supervisor says things that make me think he/she supports breastfeeding

My coworkers do not make fun of me when I sometimes leak milk through my clothes

I can easily find a quiet place other than the bathroom at work to pump breast milk

My coworkers listen to me talk about my breastfeeding experience

Technical Support dimension

My workplace has a refrigerator that I can use to store my milk

My workplace has a breast pump for nursing mothers to use

My workplace has an on-site day care

Workplace policy dimension

My job could be at risk (e.g., lose my job or get fewer scheduled hours) if I breastfed or pumped breast milk at work

I would have enough maternity leave (paid and/or unpaid time off) to get breastfeeding started before going back to work

I am certain my company has written policies for employees that are breastfeeding or pumping breast milk
